# Psoriasis and oral health in adult United States population: a cross-sectional study

**DOI:** 10.1186/s12903-022-02689-y

**Published:** 2023-02-02

**Authors:** Mai Hussein, Youssef M. K. Farag, Stephen Sonis

**Affiliations:** 1 Harvard Medical School, 25 Shattuck Street, Boston, MA 02115 USA; 2 Ministry of Health and Population, Cairo, Egypt; 3 Department of Epidemiology, Johns Hopkins Bloomberg School of Public Health, Baltimore, MD USA; 4 Division of Oral Medicine and Dentistry, Brigham and Women’s Hospital/Dana Farber Cancer Institute, Boston, MA USA; 5 Department of Oral Medicine, Infection and Immunity, Harvard School of Dental Medicine, Boston, MA USA; 6 Primary Endpoint Solutions, LLC, Waltham, MA USA

**Keywords:** Periodontitis, Psoriasis, Quality-of-life, Bone loss, Inflammation, Autoimmunity

## Abstract

**Objectives:**

To detect the association between psoriasis as an exposure and oral health conditions as outcomes represented by periodontal and dentition status. This was addressed by analysis of a large number of adults in the United States.

**Methods:**

By using The National Health and Nutrition Examination Survey datasets from 2009 to 2014, we performed a cross-sectional analysis of 11,726 participants included in our study population. For participants aged ≥ 30 years, the psoriasis status was assessed from the medical questionnaire. We used data from periodontal and oral examinations to assess the oral conditions of our participants. We examined the association between psoriasis as exposure and moderate/severe periodontitis and non-functional dentition as outcomes.

**Results:**

The weighted prevalence of psoriasis was 3%, 44% for moderate/severe periodontitis, and 20.5% for non-functional dentition. The fully adjusted model showed no significant association between psoriasis and moderate/severe periodontitis (Prevalence Ratio 1.02, 95% CI 0.9–1.2, *p* = 0.8). There was no statistically significant association between psoriasis and non-functional dentition except in the fully adjusted model it became statistically significant (Prevalence Ratio 0.8, 95% CI 0.7–0.9, *p* = 0.04).

**Conclusion:**

Our results showed no association between psoriasis and periodontal or dentition status except in a fully adjusted model for non-functional dentition.

## Introduction

Periodontitis is a common chronic inflammatory disease that destroys the teeth-supporting structure and affects about 50% of adults worldwide [1]. Alveolar bone resorption associated with periodontal disease is a major cause of tooth loss [2].

Psoriasis is an immune-mediated skin disorder that reportedly affects approximately 7.5 million American adults [3]. Malfunction in the immune system leads to the abnormal accelerated generation of skin cells resulting in the development of patchy lesions that vary in size and surface area [4]. Although it is a non-fatal chronic disease, psoriasis is associated with an increased risk of other health complications mostly psoriatic arthritis. Recently, the association of psoriasis with hypertension, obesity, type 2 diabetes, myocardial infarction, and the risk of stroke [5]. A relationship between psoriasis and periodontitis has been suggested previously [6]. Both share pathophysiologic features including an activated innate immune cell compartment with neutrophil dominance and a cytokine milieu with the presence of IL-17/tumor necrosis factor-α, an exaggerated immune response to the resident microbiota [7]. Given their shared biology, it has been suggested that periodontitis may predispose to psoriasis or, at the very least, be associated with its presence Additionally, several lifestyle behaviors associated with psoriasis are also more prevalent in participants with periodontitis [8, 9]. These include smoking, alcohol, decreased physical activity, and obesity.

While a relationship between psoriasis and periodontitis has been noted, studies assessing an association have largely relied on observational data did not assess statistical significance, and did not report an association between clinical or radiographic periodontitis parameters and psoriasis after adjustment for confounders [10–12]. In contrast, other literature showed no differences between psoriasis patients and control individuals [13]. Adjustments of risk factors such as demographics, lifestyle, and comorbidities are needed to be done to control their confounding effect. Thus, it is unclear if an association exists between periodontitis and psoriasis, and if it does, the nature of that association. Also, there is a deficiency in the studies which tried to address the association between both diseases. Consequently, in this study, we aim to assess the real association between periodontitis/dentition status and psoriasis and whether the association is sustained after adjustment for confounding factors by using a real-world data analysis from a large nationally representative sample of adults in the United States.

## Methods

*Study population* Our study population was obtained using data from the National Health and Nutrition Examination Survey (NHANES) datasets from 2009 to 2014. NHANES was a national United States (US) cross-sectional study conducted annually and the data was released every two years by the National Center for Health Statistics (NCHS) and the Center for Disease Control and Prevention (CDC). NHANES represented the non-institutionalized civilian US population in all 50 states by using a complex, multistage probability design [14]. Institutional Review Board (IRB) for NCHS reviewed and approved the NHANES protocol. Data for psoriasis and clinical oral examination for periodontal status and remaining teeth were available in the 2009–2014 cycles. These cycles contained periodontal and dentition data by full mouth clinical examination for each participant. More recent NHANES cycles did not include a periodontal examination. Our study followed the STROBE checklist.

**Fig. 1:**
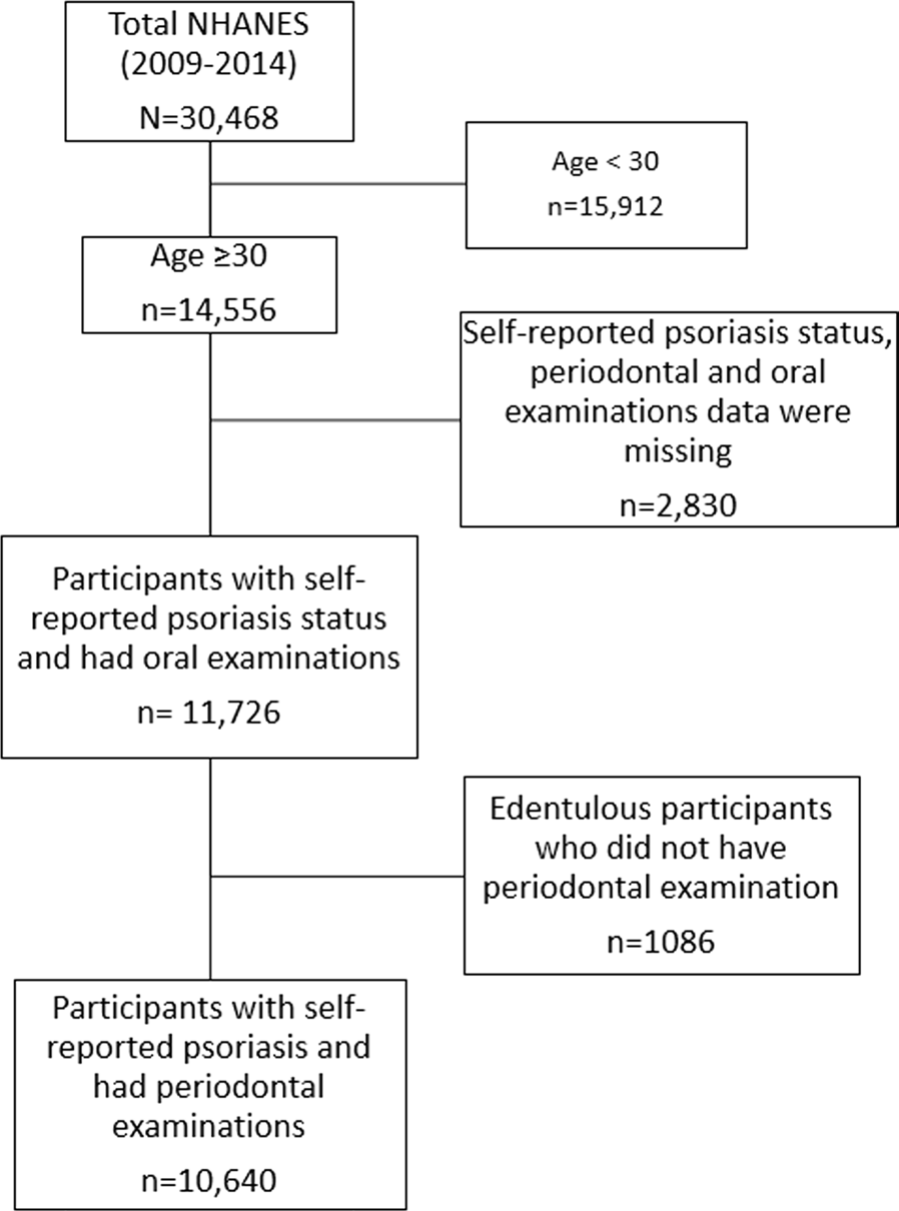
Consort diagram of the study population NHANES 2009–2014

*Inclusion and exclusion criteria* Inclusion criteria were adults ≥ 30 years of age who self-reported psoriasis status and had periodontal, dentition data, and oral examination data. We excluded participants with missing exposure or outcome data. Among 30,468 our study population included 10,640 adults who self-reported psoriasis status data and had a periodontal examination. An additional 1086 participants self-reported psoriasis status data and for whom the dentate status was known, as edentulous participants did not have a periodontal examination. So, 11,726 participants were included to evaluate the association between psoriasis and dentition (Fig. 1). Adults aged 30 years and older were eligible for a full-mouth periodontal examination according to the protocol introduced in NHANES.

### Psoriasis status

The presence and severity of psoriasis were the exposure variables. They were determined by using a medical condition questionnaire. Participants were asked to answer affirmatively or negatively whether they had ever been told by a healthcare provider that they had psoriasis. Participants were then categorized into two groups: participants with or without psoriasis. Psoriasis severity was assessed by questionnaire and reported in cycles (2011–2014). Participants were asked if they had little or no psoriasis (< 1 palm of hand), only a few patches (1 or 2 palms of hand), scattered patches (3–10 palms of hand), or extensive psoriasis (> 10 palms of hand). We categorized participants by psoriasis severity into three groups; either no psoriasis, little/few, or scattered/extensive.

### Oral conditions outcomes

*Periodontal status* We defined periodontitis by using oral examination data. Oral examination was conducted by licensed dentists in the mobile examination center (MEC). A reference examiner visits each dental examiner 2–3 times a year and conducts about 20 replicate exams during each visit. Data from these replicate exams were used to produce inter-rater reliability statistics to objectively evaluate examiner agreement. Full-mouth periodontal examination included assessment of clinical attachment level (CAL) and probing pocket depth (PPD). CAL was measured by calculating the distance from the cement-enamel junction (CEJ) to the depth of the sulcus. PPD was measured from the free gingival margin (FGM) to the depth of the sulcus by a periodontal probe. Measurements were done in six sites per tooth (distal, mid-facial, mesio-facial, distal-lingual, mid-lingual, mesio-lingual) by using World Health Organization (WHO) periodontal probe. CAL was used because it indicates the cumulative loss of support from the aggregate effects of pathologic factors. That was done for all the teeth except the third molars because of their frequent absence and malposition. Participants were classified for their periodontal status according to definitions proposed by the Centers for Disease Control and Prevention (CDC) in collaboration with the American Academy of Periodontology (AAP). We used the 2012 CDC/AAP case definition and the European Federation of Periodontology (EFP) [15]**.** We categorized participants into two groups based on their periodontal status: no/mild periodontitis (NMP) and moderate/severe periodontitis (MSP). Severe periodontitis was diagnosed when ≥ 2 interproximal sites with CAL ≥ 6 mm (not on the same tooth) and ≥ 1 inter-proximal site with PPD ≥ 5 mm. Moderate periodontitis was diagnosed when ≥ 2 interproximal sites with CAL ≥ 4 mm (not on the same tooth) or ≥ 2 interproximal sites with PPD ≥ 5 mm. Participants had neither moderate nor severe periodontitis (no/mild periodontitis). For the statistical analysis, we combined moderate and severe periodontitis (MSP) in the same group.

*Dentition status* It was detected to measure oral health status. We assessed the presence or absence of whole natural permanent teeth except the third molars using oral examination data. We categorized participants into two groups; those who had functional dentition and those who did not. Independently from teeth positions or conditions, functional dentition was defined as having ≥ 20 teeth, and non-functional dentition was defined as having ≤ 19 teeth [16].

### Other variables

Data about demographic, socioeconomic, dietary, and health-related questions were collected by interview. Medical examination, physiological measurements, and laboratory tests were administrated in MEC. Sociodemographic data included: age, gender, race/ethnicity “Non-Hispanic White, Non-Hispanic Black, Mexican American or Hispanic, or Other Race- Including Multi-Racial”, income level “ < 100% (poor), 100–199% near-poor, 200–499% (not poor), or ≥ 500% of Federal Poverty Level (FPL)”, education “less than high school, high school, or more than high school”, health insurance “private, or no/public”. The smoking status of the participants was categorized into three groups: never, former, or current [17]. Never smokers had smoked < 100 cigarettes. Former smokers had smoked ≥ 100 cigarettes. Current smokers had smoked ≥ 100 cigarettes and smoked every day or some days at the time of the survey. By using body mass index (BMI) data, we divided participants into normal (BMI < 25 kg/m^2^), overweight (BMI 25–29 kg/m^2^), or obese (BMI ≥ 30 kg/m^2^). Health insurance was categorized into no, public, and private. Diabetes was determined with questionnaire data. Participants were considered diabetic if they had ever been told by a doctor that they had diabetes, took diabetic pills or took insulin. Participants were diagnosed with hypertension if they had systolic blood pressure ≥ 130 mmHg, diastolic blood pressure ≥ 90 mmHg, self-reported hypertension, or self-reported hypertensive medication. Participants who did not know or refused to answer questions about their psoriasis status were considered missing from our analysis.

### Statistical analysis

We appended three cycles (NHANES 2009–2010, 2011–2012, and 2013–2014) to obtain the analytic dataset. We used the svy command and appropriate weights [18]. The baseline and demographic characteristics of our study sample were categorized according to the presence or absence of psoriasis. Continuous variables were presented with mean and standard error (SE). categorical variables were presented with proportions. Chi-squared was used to compare baseline characteristics between participants with and without psoriasis. We evaluated the associations by using Poisson regression models. Poisson regression models estimated the prevalence ratio (PR) and 95% confidence intervals (CI). We used poison regression analysis due to using of the survey analysis and the high prevalence of the outcome variables, 40% MSP and 20.5% non-functional dentition, to avoid possible inappropriate estimation of the association [19]. We progressively adjusted these models for different sets of potential confounders. Model 1 was the crude association. Model 2 was adjusted for demographic variables such as age, gender, race, income level, and health insurance. Model 3 was adjusted for demographics and lifestyles like smoking. Model 4 was adjusted for demographics, lifestyle, and clinical variables such as diabetes and hypertension [20, 21]. For the sensitivity analyses we estimate the association between psoriasis severity and periodontal/dentition status, we decided to stratify the overall results by age categories. We considered two-sided *p* value < 0.05 statistically significant. The analysis was done by using StataCrop LLC: Stata/IC 16.1.

## Results

There were 11,726 participants included in our study population. Three hundred and forty-two (*n* = 342) participants self-reported psoriasis, the weighted prevalence of psoriasis was 3%, 44% of severe to moderate periodontitis, and 20.5% of non-functional dentition from the whole population.

Table 1 shows the unadjusted baseline characteristics of the participants who were included in the analytic sample stratified by psoriasis prevalence status. No significant differences were seen in the prevalence of psoriasis or most baseline characteristics except for smoking status and race. Smoking status and race were significantly different according to psoriasis status prevalence. The weighted prevalence of MSP in the psoriasis group compared to those without psoriasis (45.6% vs. 44%, *p* = 0.6). Also, the weighted prevalence of non-functional dentition in the psoriasis group compared to those without psoriasis (19% vs. 20.5%, *p* = 0.6).

**Table 1:** Baseline demographics and clinical characteristics of the study population NHANES 2009–2014

Characteristics		Total population		Psoriasis*		Non-psoriasis		*p* value
		N	%	n	%	n	%	
		11,726		342	3%	11,384	97%	
Age	Mean (SE)		52 (0.3)		53.4 (0.8)		51.8 (0.3)	0.07
	30–44 years	2570	23%		19%		23%	0.4
	45–59 years	4872	47%		49.5%		47%	
	> = 60 years	4284	30%		31.5%		30%	
Gender	Male	5786	48.5%		49%		48.5%	0.9
	Female	5940	51.5%		51%		51.5%	
Race	Mexican American or Hispanic	2732	13%		9.4%		13%	0.002
	Non-Hispanic White	5148	69%		78.5%		68.5%	
	Non-Hispanic Black	2467	11%		6%		11%	
	Other Race—Including Multi-Racial	1379	7.3%		6%		7.5%	
Income level	< 100% FPL **	1862	11%		12%		11%	0.9
	100–199% FPL	2520	16.5%		16%		16.5%	
	200–499% FPL	2926	28.5%		29%		28.5%	
	> = 500% FPL	3418	44%		43%		44%	
Education	Less than high school	2990	17%		16%		17%	0.9
	High school	2581	21.5%		21%		21.5%	
	More than high school	6140	61.5%		63%		61.5%	
Health Insurance	Private	4648	50%		44.5%		50%	0.2
	Public	4534	33%		39%		33%	
	No	2517	17%		16%		17%	
BMI	Normal	3126	27%		20%		27%	0.1
	Overweight	4049	35.5%		39%		35.5%	
	Obese	4461	37.5%		41%		37.5%	
Smoking^‡^	Never	6332	54.5%		43%		55%	< 0.01
	Former	3067	27%		42.5%		26.5%	
	Current	2322	18.5%		14.5%		18.5%	
Dental need	Regular/routine care	4690	47.5%		51%		47.5%	0.3
	Need dental treatment	7036	52.5%		49%		52.5%	
Periodontitis	Moderate/Severe	5459	44%		45.6%		44%	0.6
	No/Mild	5181	56%		54.4%		56%	
Dentition	Non-functional	3296	20.5%		19%		20.5%	0.6
	Functional	8430	79.5%		81%		79.5%	

*BMI* body mass index

*Psoriasis includes the weighted percentage and population of NHANES participants who self-reported that they had psoriasis

**FPL, federal poverty level

^‡^Smoking status: never smokers were those who had smoked ≤ 100 cigarettes; former smokers were those who had smoked 100 + cigarettes in their lifetime but no longer smoked; current smokers were persons who had smoked 100 + cigarettes in their lifetime and smoked every day or some days at the time of the survey

Table 2 summarizes the Poisson regression models used to estimate our participants' association between psoriasis and periodontal and dentition statuses by measuring the PR and 95% CI.

**Table 2:** Prevalence Ratios (95% CI) of prevalent moderate/severe periodontitis for psoriasis status, NHANES 2009–2014

Psoriasis status	Moderate/severe periodontitis**			Non-functional dentition***		
	No-psoriasis	Psoriasis		No-psoriasis	Psoriasis	
		PR (95% CI)	*p* value		PR (95% CI)	*p* value
Model 1 (Unadjusted)	Ref	1.04 (0.88–1.22)	0.61	Ref	0.93 (0.73–1.18)	0.56
Model 2 (Adjusted)	Ref	1.03 (0.88–1.21)	0.72	Ref	0.87 (0.71–1.07)	0.19
Model 3 (Adjusted)	Ref	1.03 (0.88–1.19)	0.74	Ref	0.85 (0.71–1.02)	0.08
Model 4 (Adjusted)	Ref	1.02 (0.87–1.19)	0.81	Ref	0.81 (0.67–0.97)	0.02

*Model 1: Unadjusted association

*Model 2: Adjusted for demographics; age, gender, race, income level, education, and health insurance

*Model 3: Adjusted for demographics and lifestyle as smoking

*Model 4: Adjusted for demographics, lifestyle, and clinical variables; diabetes and hypertension

**Ref: No/Mild periodontitis (PR = 1)

***Ref: Functional dentition (PR = 1)

For periodontal status, the unadjusted PR and (95% CI) for MSP to compare participants with or without psoriasis were 1.04 (0.9–1.2, *p* = 0.6) which was statistically insignificant. Nearly similar results were observed with multivariable adjustment and there were not statistically significant for all models The significance of the association was lost after adjusting for potential confounders. When the model was fully adjusted (model 4), the PR for MSP comparing participants with or without RA was 1.02 (0.9–1.2, *p* = 0.8).

For dentition status, we found in model 1 that the unadjusted PR and (95% CI) for non-functional dentition to compare participants with or without psoriasis were 0.9 (0.7–1.2, *p* = 0.6). This result remained not statistically significant even after adjustment for demographics and lifestyle factors. In contrast, it became statistically significant after the multivariable fully adjusted model (Model 4), the PR for non-functional dentition comparing participants with or without psoriasis was 0.8 (0.7–0.9, *p* = 0.04).

For sensitivity analysis, Table 3 shows the Poisson regression models which estimate the association between psoriasis severity and periodontal and dentition status for psoriasis participants by measuring the PR and 95% CI. Whereas the unadjusted PR and (95% CI) for MSP (Model 1) in participants with little or few psoriasis and participants with scattered or extensive psoriasis were compared to participants without psoriasis 0.8 (95% CI 0.6–1.1, *p* = 0.2) and 0.9 (95% CI 0.5–1.7, *p* = 0.8) respectively, which were statistically insignificant. Nearly similar results were observed with multivariable adjustment and there were not statistically significant for all adjusted models.

**Table 3:** Prevalence Ratios (95% CI) of prevalent moderate/severe periodontitis for psoriasis severity, NHANES 2011–2014

	Psoriasis severity	Moderate/ severe periodontitis		Non-functional dentition	
		PR (95% CI)	*p* value	PR (95% CI)	*p* value
Model 1 (Unadjusted)	No psoriasis	Ref		Ref	
	Few/little	0.83 (0.64–1.09)	0.19	0.81 (0.57–1.15)	0.24
	Scattered/extensive	0.94 (0.52–1.69)	0.83	0.67 (0.32–1.39)	0.28
Model 2 (Adjusted)	No psoriasis	Ref		Ref	
	Few/little	0.83 (0.63–1.09)	0.18	0.76 (0.57–1.01)	0.06
	Scattered/extensive	1.01 (0.59–1.71)	0.98	0.82 (0.45–1.48)	0.50
Model 3 (Adjusted)	No psoriasis	Ref		Ref	
	Few/little	0.85 (0.65–1.12)	0.24	0.78 (0.60–1.01)	0.06
	Scattered/extensive	1.05 (0.64–1.73)	0.84	0.85 (0.48–1.51)	0.58
Model 4 (Adjusted)	No psoriasis	Ref		Ref	
	Few/little	0.84 (0.64–1.11)	0.21	0.75 (0.58–0.97)	0.03
	Scattered/extensive	1.02 (0.61–1.71)	0.93	0.72 (0.39–1.31)	0.27

*Model 1: Unadjusted association

*Model 2: Adjusted for demographics; age, gender, race, income level, education, and health insurance

*Model 3: Adjusted for demographics and lifestyle as smoking

*Model 4: Adjusted for demographics, lifestyle, and clinical variables; diabetes and hypertension

For non-functional dentition for our participants. In model 1 the unadjusted PR and (95% CI) comparing participants with little or few psoriasis and participants with scattered or extensive psoriasis to participants without psoriasis were 0.8 (0.6–1.1, *p* = 0.2) and 0.7 (0.3–1.4, *p* = 0.3) respectively, that was statistically insignificant. This result remained not statistically significant even after adjustment for demographics and lifestyle factors and clinical variables. In the fully adjusted model (Model 4), the PR for non-functional dentition comparing participants with little/few vs. no psoriasis was 0.8 (0.6–0.9, *p* = 0.03) which was statistically significant. In contrast, comparing participants with scattered/extensive psoriasis to no psoriasis the PR was 0.7 (0.4–1.3, *p* = 0.3) which became statistically insignificant.

## Discussion

By using a representative sample of the adult US population, we found there was no statistically significant association between psoriasis and the periodontal status or dentition status of our participants. Because it is a study with a large sample it could be considered as support to establish that there is no relationship between the prevalence of psoriasis and the presence of periodontal disease or high tooth loss.

Both psoriasis and periodontitis share common risk factors, dysregulating host immune response in epithelial surfaces [22–24]. Cell-mediated immune response and inflammation are initiated by the interplay between the microbiota, risk factors, and genetic factors. That leads to triggers dendritic cells (DCs) and the production of proinflammatory cytokines [25]. Although previous studies showed an increasing prevalence of periodontitis in the psoriatic population, they had some limitations. These limitations were either differences in the measurement of the periodontal status, small sample size [10–12], non-accounting of the disease severity [26], or lack the adjustments of some confounders [27]. Therefore, more research into this area is needed. Our findings were different from the previous literature, we used a different population and a larger sample size. Also, previous studies did not adjust for confounders which we did in our study. That could explain the difference in our results.

For the dentation status, although there was no association that had been found with the unadjusted model or after adjustment for demographics and lifestyle, a statistically significant association appeared with the fully adjusted model that was adjusted for demographics, lifestyle, and comorbidities. We used a dentition status to reflect the number of remaining teeth in the participants’ mouths. Participants with psoriasis have less prevalence of having non-functional dentation compared to participants without psoriasis. Most participants with psoriasis take immunosuppressive medications which have an impact on decreasing systemic inflammation. Periodontitis and alveolar bone resorption lead to tooth loss is cumulative destruction. So, one of the possible explanations could be the long duration of taking the immunosuppressive medications which affect periodontal and alveolar bone destruction. Especially, 81% of our psoriatic population was above 40 years so they were probably under medications for more than 10 years as most psoriasis cases are diagnosed at age 20–30 years old [28]. However, that also had been reflected in their dental needs as 51% needed regular dental care versus 49% needed immediate dental intervention.

A significant association was found between few/little psoriasis and non-functional dentition. Participants with few/little psoriasis were less prevalent to have non-functional dentition, but this result was not statistically significant and lost the association with participants with scattered/extensive psoriasis. However, that gives another explanation for the impact of the psoriasis severity in these associations.

The strength of our study is that we used a large sample of the adult US population and included participants of different age groups, which supported the external validity of our results and allowed for generalizability. To assess the internal validity of our study, we adjusted our models to different sets of confounders to estimate the adjusted association. That increased the precision of our results. Also, we used data from clinical periodontal assessment and oral examination to measure our outcomes. That increased the accuracy of our data. By using these methods, we tried to overcome the limitations of the previous studies. However, our study also had some limitations. We used a cross-sectional design as the exposure and outcome were measured at the same time and lacked a temporal relationship. So, we could not assess the causality between exposure and outcomes. This design lacked the temporal relationship that could not allow us to determine the periodontal history of the participants or overcome the overlap between periodontitis and tooth loss and not allow us to assess the history of psoriasis severity or its treatment. Although patients with edentulous might be due to periodontal disease. In NHANES periodontal examination was not done on edentulous patients as it depended on measuring CAL and PPD for each tooth in six sites. Also, NHANES did not collect data about the patients’ dental history. So, we did not have data about previous periodontal conditions of the edentulous patients. Part of our data was self-reported which could lead to recall bias and psoriasis severity based on the questionnaire so it might have misclassification bias. We could not account for the effect of the medications or exclusion diet as the dataset did not measure the onset or duration of the medications. We could not assess the inflammatory status by biomarkers (e.g., C-Reactive Protein CRP) as there was no data for it in the laboratory files of the selected cycles. Dentition status was assessed by the teeth present independently from their condition. Finally, there could be a possible biological reverse association between Periodontitis and psoriasis.

To overcome these limitations, prospective studies, investigating temporal sequences where the presence of psoriasis results in an increased incidence rate of periodontitis (or vice versa) are needed. Also, to account for the periodontal treatment on improving and controlling the psoriasis severity. That would help in providing a new approach for increasing awareness and focusing on periodontitis prevention for individuals with psoriasis and maintaining good oral health to control psoriasis status.

## Conclusion

Although previous studies showed an increasing prevalence of periodontitis in the psoriatic population, they had a small sample size and non-accounting for confounders which we tried to address in our study and that changed the results of the association. Our results showed no association between psoriasis and periodontal or dentition status. Further longitudinal studies would be needed to assess the question of causality between psoriasis as an autoimmune disease and oral health.

## Data Availability

Data are available upon request by mailing the first author, Mai Hussein (Mai.mk.hussein@gmail.com).
